# Determinants and outcomes of access-related blood-stream infections among Irish haemodialysis patients; a cohort study

**DOI:** 10.1186/s12882-019-1253-x

**Published:** 2019-02-26

**Authors:** Husham Mohamed, Alaa Ali, Leonard D. Browne, Nuala H. O’Connell, Liam Casserly, Austin G. Stack, Wael F. Hussein

**Affiliations:** 1 Division of Nephrology, Department of Medicine, University Hospital Limerick, Limerick, Ireland; 2 Graduate Entry Medical School, University of Limerick, Limerick, Ireland; 3 Division of Microbiology, Department of Medicine, University Hospital Limerick, Limerick, Ireland; 4 Health Research Institute, University of Limerick, Limerick, Ireland

**Keywords:** Haemodialysis, Arteriovenous fistula, Bacteraemia, Catheter infections, Catheter-related bloodstream infections, Access-related bloodstream infections, Central venous catheter

## Abstract

**Background:**

Infections are the second leading cause of death and hospitalisation among haemodialysis (HD) patients. Rates of access-related bloodstream infections (AR-BSI) are influenced by patient characteristics and local protocols. We explored factors associated with AR-BSI in a contemporary cohort of HD patients at a tertiary nephrology centre.

**Methods:**

A retrospective cohort of 235 chronic HD patients was identified from a regional dialysis programme between Jan 2015 and Dec 2016. Data on demographics, primary renal disease, comorbid conditions and dialysis access type were obtained from the *Kidney Disease Clinical Patient Management System (KDCPMS*). Data on blood cultures were captured from the microbiology laboratory. Poisson regression with robust variance estimates was used to compare infection rates and relative risk of AR-BSI according to the site and type of vascular access.

**Results:**

The mean age was 65 (± 15) years, 77% were men, and the median follow up was 19 months (IQR: 10–24 months), accumulating 2030 catheter-months and 1831 fistula-months. Overall rates of AR-BSI were significantly higher for central venous catheter (CVC) compared to arteriovenous fistula (AVF), (2.22, 95% (CI): 1.62–2.97) versus 0.11 (0.01–0.39) per 100 patient-months respectively), with a rate ratio of 20.29 (4.92–83.66), *p* < 0.0001. This pattern persisted across age, gender and diabetes subgroups. Within the CVC subgroup, presence of a femoral CVC access was associated with significantly higher rates of AR-BSI (adjusted RR 4.93, 95% CI: 2.69–9.01). Older age (75+ versus < 75 years) was not associated with significant differences in rates of AR-BSI in the unadjusted or the adjusted analysis. Coagulase negative Staphylococcus (61%) and Staphylococcus aureus (23%) were the predominant culprits. AR-BSIs resulted in access loss and hospitalisation in 57 and 72% of events respectively, and two patients died with concurrent AR-BSI.

**Conclusions:**

Rates of AR-BSI are substantially higher in CVC than AVF in contemporary HD despite advances in catheter design and anti-infective protocols. This pattern was consistent in all subgroups. The policy of AVF preference over CVC should continue to minimise patient morbidity while at the same time improving anti-infective strategies through better care protocols and infection surveillance.

**Electronic supplementary material:**

The online version of this article (10.1186/s12882-019-1253-x) contains supplementary material, which is available to authorized users.

## Background

Patients on haemodialysis (HD) endure infection rates that are more than 26 times higher than that of the general population [1], and more than 100 to 200-fold higher for specific organisms [2]. They are the second leading cause of hospitalisation and mortality in the dialysis population [3–5]. National and international guidelines along with national policy initiatives [6–9] recommend the use of arteriovenous fistula (AVF) whenever possible, as the risk of infections and other complications is highest among patients using central venous catheters (CVCs) [3, 10, 11]. Despite the dangers associated with CVC use, these devices remain the principal type of access in the majority of HD patients in Ireland [12, 13] and internationally [14].

The alarmingly high rates of access-related bloodstream infections (AR-BSI) in patients undergoing dialysis with a CVC has forced changes in clinical practices that include better anti-infective protocols, increasing adoption of catheter lock solutions, and better anti-microbial surveillance protocols in order to reduce CVC-related infection rates [15–18]. It is unclear, however, to what extent these changes have curbed the high rates of AR-BSI in the context of an increasing elderly HD phenotype with a high burden of complex health problems. It is also uncertain whether any benefit derived from these measures extends to very high-risk groups especially the elderly, patients with diabetes and those dialysed with a femoral CVC. While the formation of a functioning AVF is the preferred vascular access, this is not easily attainable in all individuals, especially elderly patients on HD [19]. Furthermore it remains controversial whether CVCs are superior to AVFs among elderly patients undergoing dialysis with a recent study finding lower rates of catheter-related bacteraemia in elderly patients compared to younger patients [18, 20–22].

Within the Irish health system, data is lacking on the on the frequency and impact of AR-BSI in HD. The availability of such data along with clinical outcomes will help inform healthcare providers and policy-decision makers on access type, and will drive quality improvement initiatives to improve patient outcomes. We determined the rates of AR-BSI in a contemporary cohort of HD patients dialysed with a CVC or AVF and explored the relative contribution of demographic and clinical factors to overall rates of AR-BSI.

## Methods

### Study design and setting

We conducted a retrospective observational study to explore AR-BSI in a contemporary cohort of HD patients. We identified all adult patients receiving chronic HD during 2015 and 2016 under the care of a tertiary nephrology centre. Patients were observed from the first to the last dialysis they received during the period between 1/1/2015 and 31/12/2016. Primary access type and changes from CVC to AVF or vice versa during the observation period were recorded. All AR-BSI events were captured during the observation period and outcomes of these events were recorded. The rates of BSIs were calculated using standard definitions described below. As this study aimed to examine rates of bacteraemia associated with access types used over prolonged periods of time in outpatient settings, temporary dialysis catheters were not included in the analysis.

#### Description of local practice

Patients received dialysis at a unit attached to the main hospital or at an affiliated outpatient dialysis unit. All tunnelled CVCs were inserted by interventional radiologists. CVC type used is ProGuide™, produced by Merit Medical Systems®. An access care bundle was in place to reduce risk of infection. This included protocols for hand hygiene and use of protective equipment during connection and disconnection of dialysis lines. BioPatch® (Ethicon©) dressings were applied to the exit site, and were changed on a weekly basis. Catheters were locked with 46.7% citrate. Disposable catheter hubs were used. Before connection, catheter hubs and fistula needle insertion sites were decontaminated with 10% iodinated povidone. When an access infection was suspected, two sets of blood cultures were taken from each port of a catheter or from a peripheral vessel in case of a fistula. Empiric antimicrobial therapy was commenced when there was strong clinical suspicion after collection of culture samples. Dialysis catheters were removed if the causative organism was *Staphylococcus aureus*, fungal, or if the infection is difficult to clear. The HD unit protocol mandates regular screening for methicillin-resistant *S. aureus* (MRSA), vancomycin-resistant Enterococcus species (VRE), extended spectrum beta lactamase (ESBL)- producing organisms and carbapenem-resistant Enterobacteriaceae (CRE). Colonization with MRSA is treated with mupirocin nasal disinfection and chlorhexidine wash for skin disinfection followed by rescreening. All patients colonized with MRSA or CRE receive dialysis in isolation rooms. All inpatient and outpatient microbiology samples from the healthcare region are sent to a single central microbiology laboratory located within the main hospital.

### Participants and data sources

Patients were identified using data from the *Kidney Disease Clinical Patient Management System (KDCPMS)*, a national multi-domain electronic health record system that tracks clinical care of HD patients in the Irish health system. Patients who received acute dialysis or holiday dialysis treatments were excluded. Study entry age, access type and access site were defined for each patient at the date of first dialysis during the study period. Baseline data were captured on age, sex, primary cause of End Stage Kidney Disease (ESKD), comorbid conditions, the type and site of the dialysis access. Blood culture results from patients during the observation period were retrieved from the microbiology laboratory database. All changes in dialysis access type and site were recorded during the study period. Access site was recorded as upper extremity or femoral location. The internal jugular vein (IJ) was the most common access at the centre, with subclavian access only reserved for situations where IJ access was not attainable. Due to the small number of non-IJ sites, comparisons between different non-femoral CVC’s was not reliable or informative. The determination of infection rates and rate ratios was based on the current access in use at time of infection.

### Definition of AR-BSI and calculation of rates

Access-related bloodstream infection (AR-BSI) was defined as growth of a typical organism with either a documented exit site or tunnel infection, or with no other identified source of infection. Patients with atypical organisms who received antimicrobial treatment for 2 weeks or more were also considered to have AR-BSI. Blood cultures that were positive with the same organism within 21 days of a previous positive culture were considered part of the initial event and not counted as a separate event. The definition of AR-BSI in our study did not include sampling from a peripheral vein (as recommended by CDC), however, previous reports suggest that peripheral blood culture results add little to the sensitivity and specificity of cultures blood obtained from the HD circuit and the venous catheter hub [23].

We followed guidelines issued by the Centers for Disease Control and Prevention (CDC) – National Healthcare Safety Network (NHSN) for calculation of event rates [24, 25]. The number of chronic HD patients under the care of the tertiary centre on the first day of each month was used as the denominator for that month. Vascular access type at the start of the month was used to identify subgroups for catheter patient-months and fistula patient-months. The numerator for AR-BSI event rates for each month was the number of identified AR-BSI events during that month. All recorded CVCs were tunnelled catheters (none were temporary dialysis catheters). Only two arteriovenous grafts were in use during the observation period. For the purposes of this analysis, these were grouped with AVF.

### Ethical approval

Ethical approval was not sought this study as the surveillance of infections in dialysis patients is part of regular clinical audit and the hospital’s quality improvement programme [26].

### Statistical analysis

Baseline characteristics were presented for the whole group and for subgroups of study entry access type. Continuous variables were presented as mean ± standard deviations and categorical variables were presented as percentages. Comparisons between groups according to vascular access type were performed by analysis of variance for continuous variables and Chi-square test for categorical variables.

Poisson regression employing the Huber-White sandwich variance estimator was used to compare the infection rates and determine the risk of infection according to vascular access type. Rates of AR-BSI were presented as events per 100 patient-months with robust 95% confidence intervals (CIs). To determine factors associated with bacteraemia among patients using CVC, univariable and multivariable models were constructed to examine the association of demographic and clinical factors, and access insertion site with the risk of AR-BSI. Model development progressed using a manual strategy taking into consideration known associations from published literature, and statistical significant univariable associations. A final model was constructed to determine the association of age, sex, diabetes, and access type with the outcome of AR-BSI in patients receiving dialysis by CVC. Goodness of fit was assessed using the Pearson and Deviance statistics. All analyses were conducted using R version 3.4.

## Results

### Baseline characteristics of study population

**Fig. 1:**
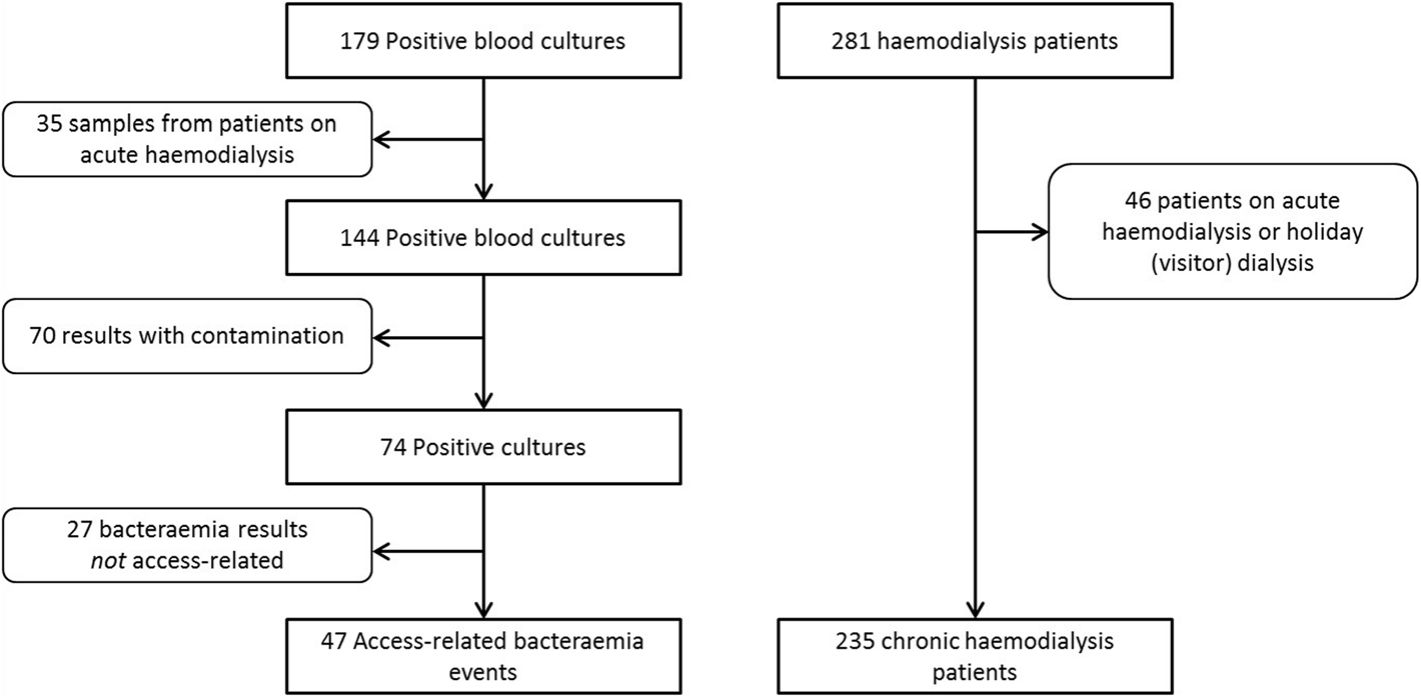
Study flow diagram

A total of 281 adult patients received dialysis between 1/1/2015 and 31/12/2016. Of those, 46 were excluded (Acute HD: 26, Visitor/Holiday HD: 20) leaving 235 patients eligible for inclusion in the final study sample. During the observation period, the monthly census increased from 134 patients to 181 from Jan 2015 to Dec 2016, and the percentage of patients receiving dialysis by CVC ranged from 48 to 57% (Additional file 1: Table S1). Median patient follow up was 19 months (IQR: 10–24 months) resulting in 3861 patient months of observation, 2030 catheter-months and 1831 fistula-months (Additional file 1: Table S2). There were 74 bloodstream infections detected during the observation period, of which 47 were related to HD access (Fig. 1).

Table 1 illustrates the baseline characteristics of participants at study entry. The average age was 65 (± 15) years, 28.5% were 75 years of age or older, and the majority were men (77%). Diabetes was the most common cause of ESKD, while hypertension and diabetes were the most prevalent recorded comorbid conditions. The distribution of baseline characteristics was similar for patients dialysed by either AVF or CVC with the exception of sex and primary cause of ESKD. The CVC group had significantly higher proportion of women and fewer patients who had diabetes, hypertension and renal cystic disease as their primary cause of renal disease.

**Table 1:** Baseline characteristics of the total population

	Overall *n* = 235❖	AVF/AVG at study entry❖ *n* = 96	CVC at study entry❖ *n* = 139
Femoral access	4.3	2.1	5.8
Age in years (mean (SD))	65 (15)	66 (14)	64 (15)
Age groups			
< 75 years	71.5	69.8	72.7
75 +	28.5	30.2	27.3
Women*	33.2	22.9	40.3
Primary cause of renal disease*			
Diabetes mellitus	23.8	27.1	21.6
Glomerulonephritis	19.6	19.8	19.4
Cystic kidney disease	8.1	13.5	4.3
Other urologic	6.4	6.2	6.5
Hypertension	3.8	6.2	2.2
Other cause	16.2	11.5	19.4
Unknown/missing	22.1	15.6	26.6
Comorbidities			
Hypertension	68.9	67.7	69.8
Diabetes	35.3	33.3	36.7
Atherosclerotic heart disease	24.3	26.0	23.0
Congestive heart failure	15.7	15.6	15.8
Other cardiac	22.1	25.0	20.1
Cerebrovascular disease	11.9	13.5	10.8
Peripheral vascular disease	8.5	7.3	9.4

❖Column % unless specified otherwise

*p < 0.05

The baseline characteristics of patients dialysed through a CVC is shown in Table 2. Patients age ≥ 75 years had significantly more hypertension and congestive heart failure than younger counterparts while the distribution of other characteristics was similar. Distribution of baseline characteristics in the whole group (any access) by age group is shown in Additional file 1: Table S3.

**Table 2:** Baseline characteristics by age group among patients with a tunnelled central venous catheter at study entry

Characteristic	All patients ❖ (*n* = 139)	Age groups (years)❖	
		< 75 (*n* = 101)	75+ (*n* = 38)
Femoral access	5.8	5.9	5.3
Age in years (mean (SD))*	64 (15)	57 (12)	82 (5)
Female	40.3	38.6	44.7
Primary cause of renal disease			
Diabetes mellitus	21.6	20.8	18.4
Glomerulonephritis	19.4	19.8	18.4
Cystic kidney disease	4.3	5.0	2.6
Other urologic	6.5	6.9	5.3
Hypertension	2.2	3.0	0.0
Other cause	19.4	19.8	18.4
Unknown/missing	26.6	24.8	31.6
Comorbidities			
Hypertension*	69.8	62.4	89.5
Diabetes	36.7	34.7	42.1
Atherosclerotic heart disease	23.0	18.8	34.2
Congestive heart failure*	15.8	10.9	28.9
Other cardiac	20.1	15.8	31.6
Cerebrovascular disease	10.8	8.9	15.8
Peripheral vascular disease	9.4	9.9	7.9

❖Column % unless specified otherwise

*p < 0.05

### Rates of access-related bloodstream infection (AR-BSI) in CVC versus AVF

**Fig. 2:**
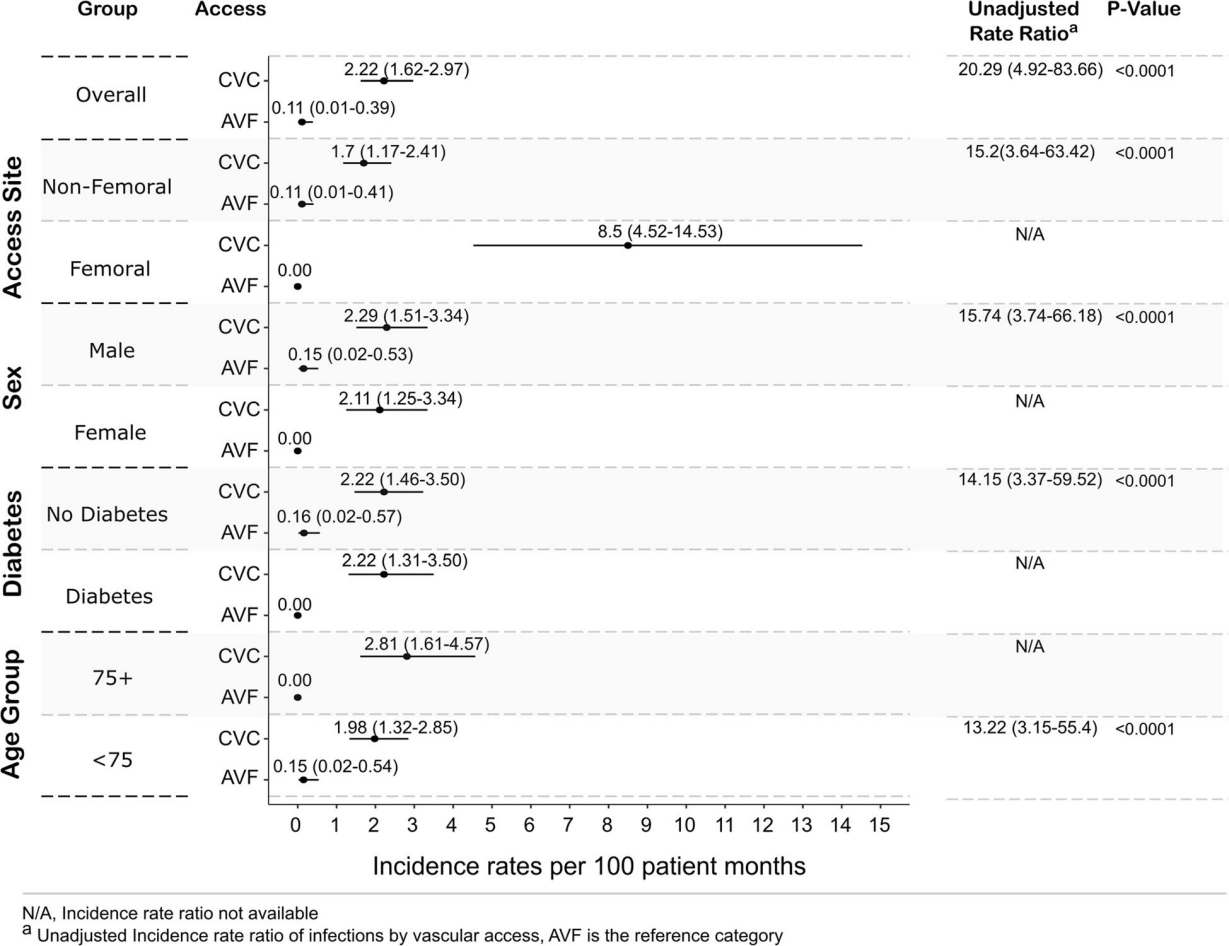
Rates of access-related bloodstream infections by access type. CVC: Central venous catheter, AVF: Arteriovenous fistula

The rate of AR-BSI in patients dialysed with a CVC was significantly higher than in patients with an AVF [2.22 (95% 1.62–2.97) versus 0.11 (95% CI: 0.01–0.39) per 100 patient months respectively, *p* < 0.001], with an unadjusted incidence rate ratio of 20.29 (95% CI 4.92–83.66) as shown in Fig. 2*.* Among all specified subgroups of age, sex, diabetes and access site, the incidence rates of AR-BSIs were significantly and substantially higher among those using a CVC compared to AVF.

**Fig. 3:**
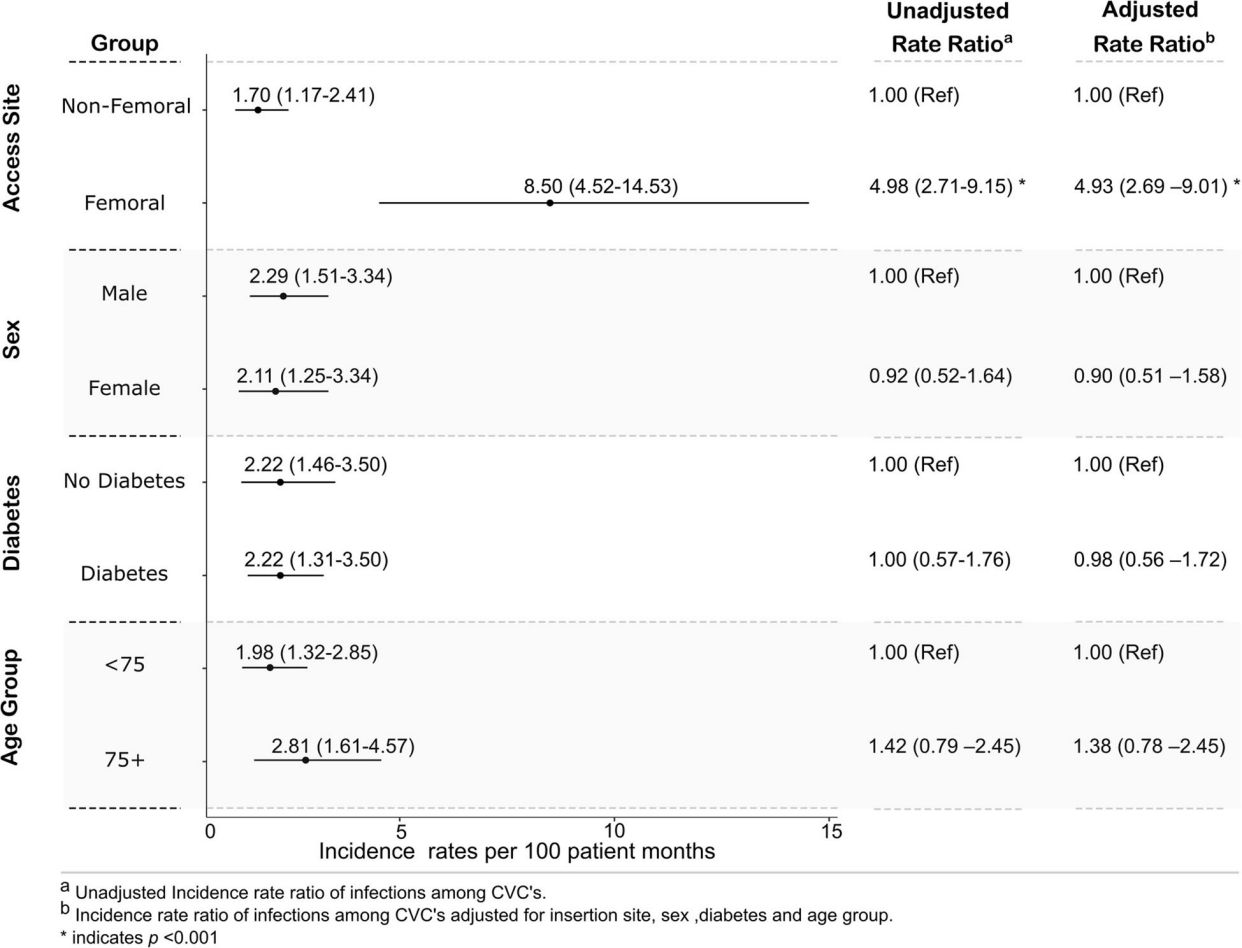
Rates and rate ratios of access-related bloodstream infection events among patients receiving dialysis by tunnelled central venous catheter. * *p* < 0.001. Multivariable model was adjusted for site of insertion, sex, diabetes and age group

By far the highest rate of AR-BSI was observed with a femoral CVC access, 8.50 (95% CI 4.52–14.53) events per 100 patient months. Among patients receiving dialysis by tunnelled catheter, the rate ratio of AR-BSI for femoral versus non-femoral CVC was 4.98 (95% CI 2.71–9.15), *p* < 0.001. In multivariable analysis adjusting for age, sex, and diabetes, use of a femoral CVC was associated with 4.93 times (95% CI 2.69–9.01) the risk of infection compared to a non-femoral CVC site – Fig. 3*.*

There were no significant differences in the rates of AR-BSI by age (75+ versus < 75 years) in the unadjusted or the adjusted analyses.

#### Type of organism

The distribution of organisms isolated from blood cultures is shown in Table 3. The most common isolates were Staphylococci identified in 85% of positive blood cultures. The majority of these were Coagulase-negative Staphylococcal (CoNS) infections with *S. aureus* contributing 23.4%. No fungal infections were recorded during the study period.

**Table 3:** Types of organisms isolated in access-related bloodstream infections

Organism	Frequency (%)
Coagulase-negative Staphylococcus	61.7
Staphylococcus aureus	23.4
MSSA: 19.1%	
MRSA: 4.3%	
Beta-haemolytic Streptococci	4.3
Streptococcus parasanguinis	2.1
Enterococcus spp.	2.1
Vancomycin-resistant enterococci (VRE)	2.1
Proteus spp.	2.1
Poly-microbial	2.1

#### AR-BSI outcomes

AR-BSIs resulted in hospitalisation and access loss in 34 (72%) and 27 (57%) of the events respectively. Two patients died with AR-BSI events; one with poly-microbial infection.

#### Sensitivity analysis

To address whether a number of individuals prone to risk in the CVC group may be leading to an exaggeration of risk, we conducted a sensitivity analysis by excluding patients with more than 1 and more than 2 recorded CRBSIs (Catheter-Related Bloodstream Infections). No variables were found to be statistically significant when excluding patients with more than 1 case of CBRSI (*n* = 10). However, excluding patients with more than two cases (*n* = 2) yielded similar results to that of the primary analysis. The median duration between CRBSI’s among these patients was 117 days with a minimum of 50 days and a maximum of 465 days between events. There were a few patients with recurrent infections (more than 2 cases of CBRSIs) in this study and omission of these patients did not alter the primary findings of this study.

## Discussion

In this large centre-based study, we emphasise the significant risk of bloodstream infections associated with use of tunnelled dialysis catheters. Compared to patients who were using an AVF, patients with a CVC experienced a 20-fold higher risk of access-related bacteraemia. The risk associated with CVC use was independent of age and comorbid disease measured at baseline. Subgroup analysis confirmed that the pattern of risk from CVC was present in younger and in older patients, men and women, and in patients with and without diabetes. These results would suggest that despite advances in anti-infective protocols, innovative catheter designs, and the implementation of national guidelines, CVCs remain a major source of serious morbidity in HD patients.

The adverse impact of CVC over AVF on catheter-related bacteraemia rates was overwhelmingly apparent from this analysis. Our observed rates of AR-BSI events were 2.22 and 0.11 events per 100 patient-months for CVC and AVF respectively, a 20-fold difference. Our findings are concordant with reports from other parts of the world. AR-BSI rates in patients with CVC and AVF were 3.1 and 0.6, and 3.5 and 1.7 per 100 patient-months from Greece and Brazil respectively [4, 18]. A study from the National Healthcare Safety Network (NHSN) in the US reported pooled rates of 2.55 and 0.23 events per 100 patient-months for CVC and AVF respectively from 2007 and 2011 [27], while a more recent report suggested improvements with estimates of 1.83 and 0.16 for CVCs and AVF respectively [24]. The patient-months distribution in this last report was 19% for CVC and 63% for AVF, reflecting a much lower dependence on tunnelled catheters than in our Irish cohort. The rates of AR-BSI in our cohort compare favourably with those from the CDC report in the US [24] in that rates were at the 50th percentile for CVC-related BSIs, and below the 25th for AVF-related infections. Despite these reassuring statistics, there is emerging evidence that further improvements are possible. Hymes et al, reported significant reductions in AR-BSI to 0.67 per 100 patient-months with the introduction of antimicrobial barrier caps [28]. A further study by the CDC Dialysis BSI Prevention Collaborative, demonstrated a sustained reduction in CVC-related BSI’s from 2.26 to 1.08 events per 100 patient-months using a bundle of BSI-preventative interventions [29]. These encouraging findings suggest that there is further scope to reduce infection rates associated with CVC use and emphasise the need for sustained quality improvement initiatives.

Controversy currently exists as to whether tunnelled dialysis catheters should be considered a satisfactory access type for dialysis in older patients [30]. A lower rate of complications in older patients would support this approach. In support of this hypothesis, Murea et al found lower rates of catheter-related bacteraemia in patients above 75 years versus younger patients (1.67 versus 5.99 events per 100 patient-months respectively, HR 0.33 (95% CI 0.20–0.55) [17], citing lower rates of nasal colonisation, less sweating, and less mechanical stress on the catheter as potential reasons. Wang et al showed similar results [16]. However, several studies have found no association between age and AR-BSIs [18, 20–22]. Furthermore, mortality risks (infection-related, cardiovascular-related, and all-cause) are higher in patients on dialysis by CVC even in elderly patients [31, 32]. The findings from our study are in direct contrast with those of Murea et al in that elderly patients experienced risks that were similar to those of younger patients.

The most significant factor associated with increased catheter-related BSIs in our population was site of the tunnelled dialysis catheter. In univariate and multivariable analysis, femoral access was associated with a fivefold increase in the rate of AR-BSI when compared to non-femoral access. This observation may relate to greater levels of skin contamination at the femoral area, relatively more difficult access for cleaning and observation, or may relate to some patient characteristics such as vintage or poor health. Femoral access is known to have higher rates of complications overall, including infection and malfunction [16, 17]. Quality improvement programs need to focus on this high-risk group of patients. We did not observe a difference in AR-BSI rates between patients with and without diabetes in univariate or multivariable analysis. Diabetes was found to be a risk factor for bacteraemia in some [16, 18] but not all studies [17]. Similarly, gender did not have an effect on infection rates, and this is consistent with multiple prior studies [16–18].

Gram positive organisms were the predominant microbes from positive blood cultures in our cohort, with only a smaller proportion of AR-BSIs attributable to Gram negative (GN) organisms. Whereas studies from the US, Brazil, Greece and Singapore reported GN bacterial growth in 15 to 26% of positive cultures [17, 18, 24, 27, 33, 34], GN bacteria were identified in less than 5% of specimens in our study. CoNS were identified in more than 60% of cases, which is a higher rate than compared to published literature. CoNS, despite being common constituents of the normal flora of the skin, can be major nosocomial pathogens and cause significant morbidity in patients with CVCs [35]. Their spread is facilitated by poor hand hygiene and inadequate disinfection or sterilisation of instruments or surfaces [35]. It is difficult however to compare frequencies between different studies because of different study inclusion and exclusion criteria, and different definitions used. Our study also highlights the high burden of these events on both the patient and the health system. Two bacteraemia-related fatalities were identified. The majority of patients with AR-BSIs required hospitalisation, and catheter replacement was required in more than 50% of patients.

A few limitations are worth mentioning. The study was retrospective in design and thus not all known risk factors were measured at baseline. Patients receiving dialysis by CVC may be inherently different to those with AVF. Our data did not enable us to characterise patients beyond the variables used in our models. We did not capture exit-site and tunnel infections in our study. However, it should be noted that there is subjectivity in the definition of these events and these may or may not be associated with bacteraemia. The study reflects a single centre experience, which may limit generalisability. In addition, we did not differentiate between incident or prevalent HD patients in our study. Therefore, we must acknowledge we were unable to assess whether dialysis/ catheter vintage modifies the relationship with infection risk in those with CVC’s. Finally, our unit’s policy on management of access-related bacteraemia did not have a standardised protocol to check for clearance of bacteraemia prior to or shortly after discontinuation of the antimicrobial agent. This precluded conducting a reliable comparison of clearance duration. These limitations, however, were counterbalanced by several strengths. *First*, our study included all chronic HD patients who received dialysis at a large centre, and none of the patients had missing data. *Second*, all microbiology test results were available from a single central laboratory ensuring consistency and reliability of reporting. This was of concern in data reported from dialysis facilities in the US, as blood cultures were analysed at several different laboratories, particularly for samples obtained after hospitalisation [24, 36]. We reported on BSI, a measure that is based on an objective test, using standard definitions. Finally, the period of follow-up was long relative to other published studies in the literature.

## Conclusions

AR-BSI remain a significant complication particularly among contemporary cohorts of patients undergoing haemodialysis by tunnelled catheters. The risk is present for all subgroups including the elderly. Access-related bloodstream infections impose a huge burden on patients and on health systems. Active surveillance of BSI linked to quality improvement initiatives should remain an integral part of all dialysis programmes to reduce catheter- associated infections and improve patient outcomes.

## Additional file

Additional file 1: **Table S1.** Number of patients and type of access in each month in the observation period. **Table S2.** Number of patient months in total and in 2015, 2016 by access type. **Table S3.** Baseline characteristics by age group in the whole study population. (DOC 83 kb)

## Abbreviations

AR-BSI: Access-related bloodstream infections
AVF: Arterio-venous fistula
CDC: Centers for Disease Control and Prevention
CI: Confidence intervals
CoNS: Coagulase-negative Staphylococci
CRBSIs: Catheter-related Bloodstream Infections
CRE: Carbapenem-resistant Enterobacteriaceae
CVC: Central venous catheter
ESBL: Extended spectrum beta lactamase
ESKD: End-stage kidney disease
GN: Gram-negative
HD: Haemodialysis
IQR: Inter-quartile ranges
KDCPMS: Kidney Disease Clinical Patient Management System
MRSA: Methicillin-resistant Staphylococcus aureus
NHSN: National Healthcare Safety Network
RR: Risk ratio
VRE: Vancomycin-resistant Enterococcus species
