# Supplementary material for: Determinants and outcomes of access-related blood-stream infections among Irish haemodialysis patients; a cohort study
Source: BMC Nephrol. 2019 Feb 26;20:68. doi: 10.1186/s12882-019-1253-x (PMC6390569; doi:10.1186/s12882-019-1253-x)
Supplement: Supplementary file 1 — Table S1. Number of patients and type of access in each month in the observation period. Table S2. Number of patient months in total and in 2015, 2016 by access type. Table S3. Baseline characteristics by age group in the whole study population. (DOC 83 kb) [file 12882_2019_1253_MOESM1_ESM.doc]

**Additional file 1: Table S1**: Number of patients and type of access in each month in the observation period.

| Month | n | AVF (%) | CVC (%) |
| --- | --- | --- | --- |
| 2015-01 | 134 | 50 | 50 |
| 2015-02 | 139 | 52 | 48 |
| 2015-03 | 140 | 51 | 49 |
| 2015-04 | 142 | 49 | 51 |
| 2015-05 | 146 | 51 | 49 |
| 2015-06 | 148 | 51 | 49 |
| 2015-07 | 151 | 51 | 49 |
| 2015-08 | 150 | 48 | 52 |
| 2015-09 | 152 | 49 | 51 |
| 2015-10 | 158 | 46 | 54 |
| 2015-11 | 157 | 46 | 54 |
| 2015-12 | 161 | 45 | 55 |
| 2016-01 | 161 | 46 | 54 |
| 2016-02 | 166 | 47 | 53 |
| 2016-03 | 167 | 47 | 53 |
| 2016-04 | 170 | 46 | 54 |
| 2016-05 | 170 | 47 | 53 |
| 2016-06 | 174 | 47 | 53 |
| 2016-07 | 177 | 47 | 53 |
| 2016-08 | 175 | 47 | 53 |
| 2016-09 | 178 | 46 | 54 |
| 2016-10 | 182 | 46 | 54 |
| 2016-11 | 182 | 45 | 55 |
| 2016-12 | 181 | 43 | 57 |

**Additional file 1: Table S2**: Number of patient months in total and in 2015, 2016 by access type

| Access | 2015 | 2016 | Total |
| --- | --- | --- | --- |
| CVC | 908 | 1122 | 2030 |
| AVF/AVG | 870 | 961 | 1831 |
| Total | 1778 | 2083 | 3861 |

(53% CVC, 47% AVF)

**Additional file 3: Table S3**: Baseline characteristics by age group in the whole study population

| **Characteristic** | **All patients** ❖  (n = 235) | **Age groups (years)** ❖ | |
| --- | --- | --- | --- |
|  |  | **<75**  (n = 168) | **75+**  (n = 67) |
| Tunnelled catheter at study entry | 59.1 | 60.1 | 56.7 |
| Femoral access | 4.3 | 4.8 | 3.0 |
| Age in years (mean (SD)) ** | 65 (15) | 58 (12) | 81 (4) |
| Female | 33.2 | 33.3 | 32.8 |
| **Primary cause of renal disease** |  |  |  |
| Diabetes mellitus | 23.8 | 24.4 | 22.4 |
| Glomerulonephritis | 19.6 | 21.4 | 14.9 |
| Cystic kidney disease | 8.1 | 9.5 | 4.5 |
| Other urologic | 6.4 | 6.5 | 6.0 |
| Hypertension | 3.8 | 3.6 | 4.5 |
| Other cause | 16.2 | 14.9 | 19.4 |
| Unknown/missing | 22.1 | 19.6 | 28.4 |
| **Comorbidities** |  |  |  |
| Hypertension | 68.9 | 67.3 | 73.1 |
| Diabetes | 35.3 | 35.1 | 35.8 |
| Atherosclerotic heart disease | 24.3 | 22.0 | 29.9 |
| Congestive heart failure** | 15.7 | 10.7 | 28.4 |
| Other cardiac | 22.1 | 19.0 | 29.9 |
| Cerebrovascular disease | 11.9 | 10.7 | 14.9 |
| Peripheral vascular disease | 8.5 | 7.7 | 10.4 |

❖ Column % unless specified otherwise

* p < 0.05, ** p < 0.01
